# Supplementary material for: Intraindividual variation of dose parameters in oncologic CT imaging
Source: PLoS One. 2021 Apr 23;16(4):e0250490. doi: 10.1371/journal.pone.0250490 (PMC8064522; doi:10.1371/journal.pone.0250490)
Supplement: Attachment — Submitted filename: Response to Reviewers.docx [file pone.0250490.s001.docx]

Isabel Lange

Medizinische Hochschule Hannover

Carl-Neuberg-Straße 1

30625 Hannover

Germany

To the Editors

*PLOS ONE Journal*

January 6^th^, 2021

**Rebuttal Letter**

Dear Dr. Chojniak,

We thank you and the reviewers for a thorough reading and constructive criticism of our manuscript and for the opportunity to revise and resubmit.

We are pleased to submit the improved research article titled “Intraindividual variation of dose parameters in oncologic CT imaging” for your consideration of publication in *PLOS ONE*.

On the following page, you will find our response to the editor and reviewer comments.

On behalf of my co-authors, I thank you for your consideration of this resubmission. We appreciate your time and look forward to your response.

Sincerely,

Isabel Lange, MD

Corresponding author

[isabel.lange@med.uni-jena.de](mailto:isabel.lange@med.uni-jena.de)

Babak Alikhani, [babak.alikhani@diakovere.de](mailto:babak.alikhani@diakovere.de)

Frank Wacker, wacker.frank@mh-hannover.de

Hans-Juergen Raatschen, Raatschen.Hans-Juergen@mh-hannover.de

Editor Comments:

1. Please ensure that your manuscript meets PLOS ONE's style requirements, including those for file naming. The PLOS ONE style templates can be found at

<https://journals.plos.org/plosone/s/file?id=wjVg/PLOSOne_formatting_sample_main_body.pdf> and <https://journals.plos.org/plosone/s/file?id=ba62/PLOSOne_formatting_sample_title_authors_affiliations.pdf>

Reply of the authors: We have considered and applied all style and file naming requirements by PLOS ONE for this submission.

2. PLOS requires an ORCID iD for the corresponding author in Editorial Manager on papers submitted after December 6th, 2016.

Reply of the authors: The corresponding author has created an ORCID iD: [https://orcid.org/0000-0002-4069-6634](https://orcid.org/0000-0002-4069-6634)

Reviewer Comments:

1- Regarding the study population, consider if it is not interesting to know how many patients were excluded from the analysis in the period evaluated. Consider using a flow diagram.

Reply of the authors: We have created a flow diagram that presents additional information about the process of filtering the raw database to find the study population included in this study. The figure can be found under Material and Methods.

2- In the discussion, describe other limiting factors of the study, such as retrospective analysis and use of different tomographic equipment, since they are potential confounders and effect modifiers.

Reply of the authors: This limitation has been added and is further elaborated on in our discussion.
